# Colonic volume in patients with functional constipation or irritable bowel syndrome determined by magnetic resonance imaging

**DOI:** 10.1111/nmo.14374

**Published:** 2022-04-05

**Authors:** Mette Winther Klinge, Klaus Krogh, Esben Bolvig Mark, Asbjørn Mohr Drewes, Lau Brix, Christin Isaksen, Milda Dedelaite, Jens Brøndum Frøkjær, Lotte Vinskov Fynne

**Affiliations:** ^1^ 11297 Department of Hepatology and Gastroenterology Aarhus University Hospital Aarhus Denmark; ^2^ Diagnostic Centre Silkeborg Regional Hospital Silkeborg Denmark; ^3^ 53171 Mech‐Sense Department of Gastroenterology and Hepatology Aalborg University Hospital Aalborg Denmark; ^4^ Department of Radiology Diagnostic Centre Silkeborg Regional Hospital Silkeborg Denmark; ^5^ 53171 Department of Radiology Aalborg University Hospital Aalborg Denmark; ^6^ King´s College Hospital NHS Foundation Trust London UK

**Keywords:** colonic transit time, colonic volume, functional constipation, irritable bowel syndrome, magnet resonance imaging

## Abstract

**Background:**

Functional constipation (FC) and irritable bowel syndrome constipation type (IBS‐C) share many similarities, and it remains unknown whether they are distinct entities or part of the same spectrum of disease. Magnetic resonance imaging (MRI) allows quantification of intraluminal fecal volume. We hypothesized that colonic volumes of patients with FC would be larger than those of patients with IBS‐C, and that both patient groups would have larger colonic volumes than healthy controls (HC).

**Methods:**

Based on validated questionnaires, three groups of participants were classified into FC (*n* = 13), IBS‐C (*n* = 10), and HC (*n* = 19). The colonic volume of each subject was determined by MRI. Stool consistency was described by the Bristol stool scale and colonic transit times were assessed with radiopaque makers.

**Key Results:**

Overall, total colonic volumes were different in the three groups, HC (median 629 ml, interquartile range (IQR)(562–868)), FC (864 ml, IQR(742–940)), and IBS‐C (520 ml IQR(489–593)) (*p* = 0.001). Patients with IBS‐C had lower colonic volumes than patients with FC (*p* = 0.001) and HC (*p* = 0.019), but there was no difference between FC and HC (*p* = 0.10). Stool consistency was similar in the two patient groups, but patients with FC had longer colonic transit time than those with IBS‐C (117.6 h versus 43.2 h, *p* = 0.019).

**Conclusion:**

Patients with IBS‐C have lower total colonic volumes and shorter colonic transit times than patients with FC. Future studies are needed to confirm that colonic volume allows objective distinction between the two conditions.

## Graphical abstract

Assessed with magnetic resonance imaging (MRI), patients suffering from irritable bowel syndrome with constipation had smaller colonic volume than healthy controls and patients with functional constipation. The difference was most pronounced in the ascending colon. MRI holds promise in evaluation of intestinal volume in colonic disease, however larger studies are needed before clinical use.

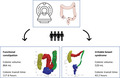

**Abbreviations**
BSFS: bristol stool form scale
FC: functional constipation
GSRS: gastrointestinal symptom rating scale
HC: healthy controls
IBS: irritable bowel syndrome
IBS‐C: irritable bowel syndrome constipation type
IQR: Interquartile range
MRI: magnetic resonance imaging
PAC SYM: patients assessment of constipation symptoms

**Key Points**

- Patients with functional constipation (FC) and patients with irritable bowel syndrome, constipation type (IBS‐C) share some symptoms. We hypothesized that assessment of colonic volume in magnetic imaging; patients with FC has a larger colonic volume than healthy controls and patients with IBS‐C.
- Patients with IBS‐C had a smaller colonic volume than either healthy controls or patients with FC.
- MRI holds promise in evaluation of intestinal volume in colonic disease, however larger studies are needed before clinical use.

## 1 INTRODUCTION

Functional constipation (FC) and irritable bowel syndrome (IBS) are common conditions, and consequences for the quality of life of those affected can be severe.^1^, ^2^ FC and IBS‐C share many similarities, and even though pain is more prominent in IBS‐C, it remains unknown whether they are distinct entities or part of the same spectrum of disease.^3^, ^4^, ^5^, ^6^ Both diagnoses are symptom‐based and highly prevalent. Depending on the definition used, 2–27% of the adult population has FC, and 7–21% has irritable bowel syndrome.^4^, ^6^, ^7^, ^8^ FC and IBS patients can be categorized by the ROME IV criteria.^2^, ^5^, ^9^

Objective evaluation of patients with FC or IBS‐C usually includes assessment of colonic transit times with, for example, radiopaque markers.^10^ The test is easy to perform and widely available. Unfortunately, many patients have normal colonic transit time despite severe symptoms, and to our best knowledge, it is not known whether the colon transit time distinguishes between FC and IBS‐C. The discrimination between the two conditions is important for handling treatment and prediction of complications. Hence, new objective methods for the evaluation of colonic function are needed. Imaging of the colon provides information on structural properties, including total and segmental colonic volumes. Ultrasonography is fast, safe, and without discomfort for the subject. Usually, the diameter of well‐defined points along the colorectum is taken as a surrogate for colonic volume, and in children, the diameter of the rectum is a useful objective marker of constipation.^11^ However, ultrasonography of the colon is observer‐dependent and does not provide a global assessment of the organ volume. CT‐scan of the colon allows a detailed description of colonic volume and content but exposes the subject to ionizing radiation.^12^

Magnetic resonance imaging (MRI) can provide safe and radiation free quantification of total and segmental colonic volumes and content without the use of contrast‐enhancing agents or bowel preparation.^13^ Radiation‐free examination is an advantage in FC and IBS, especially since many patients are women at the childbearing age. Moreover, semi‐automatic detection techniques reduce the time for otherwise comprehensive data analysis. Earlier studies have concluded that patients with constipation have larger colonic volumes than HC.^14^, ^15^, ^16^ Also, a previous study found that patients with FC had larger colonic volume than patients with IBS‐C, especially within the ascending colon.^17^ In the present explorative study, we hypothesized that MRI assessed colonic volumes of patients with FC were larger than those of patients with IBS‐C and that both patient groups have larger colonic volumes than healthy controls (HC). We, therefore, aimed to compare total and segmental colonic volumes and colonic transit times in patients with FC or IBS‐C.

## 2 MATERIALS AND METHODS

### 2.1 Subjects

Between August 2018 and November 2019, 13 patients with FC, 10 patients with IBS‐C, and 19 with HC were included. Adult patients with FC or IBS‐C were included from outpatient clinics at the Diagnostic Centre, Silkeborg Regional Hospital or the Department of Hepatology and Gastroenterology, Aarhus University Hospital, Denmark, by two experienced gastroenterologists (LF and KK). Before enrollment, they had a physical examination and medical history evaluation to ensure that they met the ROME IV criteria for FC or IBS‐C.^5^ HC were recruited through public advertising or among hospital staff. No known gastrointestinal disease and normal bowel function were requirements for inclusion of HC. Exclusion criteria for all three groups were as follows: age below 18 years, comorbidity or concomitant medication affecting gastrointestinal function, a pacemaker or neurostimulator in situ, non‐removable metallic objects, claustrophobia, and previous abdominal surgery apart from minor procedures such as appendectomy.

The study was conducted according to the Helsinki declaration and approved by the Ethical Committee, Region Middle, Denmark (1‐10‐72‐146‐17).

### 2.2 Magnetic Resonance Imaging

All participants had an MRI scan performed after 6 h fast for food and liquids. All laxatives were paused for at least 6 days before the MRI examination. Rescue medication was allowed in case of severe symptoms and noted in a special patient file. The MRI scans were performed at the Department of Radiology, Silkeborg Regional Hospital, Denmark, using a Siemens Avanto‐fit 1.5 Tesla MRI System (Siemens Healthineers, Germany). One Coronal imaging series was taken using T2‐weighted Half‐Fourier‐Acquired Single‐shot Turbo spin Echo (HASTE) with TE = 0.92 milliseconds, TR = 1200 milliseconds, flip angle = 180°, in‐plane resolution 1.6406 x 1.6406 mm, and slice thickness 4 mm, without fat saturation. Scans lasted approximately 20 s and were performed during a single breath‐hold. The participant spent approximately 5–10 min in the scanner. Each scan produced 35 to 40 contiguous images covering the entire colon, rectum, and with a resolution of 256 x 256 pixels.

### 2.3 Assessment of colonic volumes

Semi‐automatic software (Colometry v 1.0 Mech‐Sense, Aalborg University Hospital, 2015, Aalborg, Denmark) was applied to determine total and segmental colorectal volumes. Details about software performance and inter‐observer reliability have been described earlier.^18^ Regions of interest were manually defined on the T2‐weighted MRI scan. The regions included the colonic segments in each of the 35–40 coronal slices. The exact boundaries of the outer colonic surface were determined by the software based on the colonic lumen and gut wall appearing dark while fat within the adjacent organs and tissue have a brighter signal. Hence, the colonic volume measure included colonic gas, feces, and the gut wall.

The segmental colorectal volumes were divided into the cecal/ascending colon including the hepatic flexure, the transverse colon, the descending colon including the splenic flexure and the rectosigmoid. The transition between the descending colon and the rectosigmoid colon was defined by the computer software by drawing a horizontal line intersecting the anterior superior iliac pelvic crest. The distal limitation of the rectosigmoid was defined by the beginning of the anal canal. The computer‐made delineation of segments of interest was controlled by an experienced observer (MD) during a procedure lasting approximately 20 min. The observer was blinded to the group of the subject under study.

### 2.4 Questionnaires

All participants filled‐in the following questionnaires:

Bowel symptoms were described from the Bristol Stool Form Scale (BSFS), Patients Assessment of Constipation Symptoms (PAC SYM), and the Gastrointestinal Symptom Rating Scale (GSRS).

BSFS can be used as a proxy for colonic transit times and it is often used as an objective measure of constipation.^19^ Participants are asked to categorize their stools according to a pictogram showing the stool consistency and scored from 1–7, from hard separate lumps (1) to watery/liquid consistency without solid pieces (7).^19^, ^20^

PAC SYM is designed to evaluate constipation symptoms, containing 12 items divided into 3 subscales; stool symptoms, rectal symptoms, and abdominal symptoms.^21^ A high score indicates symptoms of constipation.

GSRS is a 15 items questionnaire with a 1–7‐point Likert scale developed to evaluate gastrointestinal symptoms (1 means no symptoms, and 7 means severe symptoms).^22^

### 2.5 Radiopaque markers

Whole gut and segmental colonic transit times were determined by radiopaque markers. In short, patients ingested a capsule with 10 radiopaque markers each morning for 6 consecutive days. The markers were counted on a plain abdominal x‐ray taken on Day 7. The whole gut transit time was computed as (total number of markers+5)/10, and segmental transit time calculated as number of markers in a given segment/10.^23^

Only patients with FC and IBS‐C had this examination performed.

### 2.6 Data analysis and statistics

The study was explorative and no formal power calculation was possible. Normality was checked by QQ‐plots and box plots. Kruskal–Wallis one‐way analysis of variance was applied to compare colonic volumes in the three groups. Spearman correlation analysis was used to estimate the correlation between two variables. Findings from questionnaires and segmental volumes were compared using paired t‐tests for parametric data and Wilcoxon Mann–Whitney U test for non‐parametric data. *p* < 0.05 was considered significant.

Statistical analyses were performed using Stata statistical software version 2013 (StataCorp LLC, College Station Texas, TX, USA). Graphic illustrations made by Prism 8 (GraphPad Software, San Diego, CA, USA).

## 3 RESULTS

### 3.1 Demographics

We included 13 patients with FC, 10 with IBS‐C, and 19 HC, with a combined age 19.2–64.6 (median 27.1) years. Demographic data are shown in Table 1. Patients with FC were generally older (median 45.3 years) than patients with IBS‐C (median 28.9 years) and HC (median 25.4 years). Only HC and patients with FC had a significant difference in age (*p* = 0.02). All participants were able to pause laxatives and any other medication affecting gastrointestinal function for at least 6 days before MRI and while taking radiopaque markers to assess colorectal transit time.

**TABLE 1:** Demographic data in medians and interquartile range (IQR).

	Healthy controls	Functional constipation	Irritable bowel syndrome, constipation type
Participants (*n*)	19	13	10
Females	12(63%)	11(85%)	8(80%)
Age, median (IQR)	25 (25–32)	45 (29–53)	28 (25–38)
Body mass index (kg/m^2^), median (IQR)	24 (23–26)	25 (22–31)	21.5 (19–23)
Bristol stool scale, median (IQR)	4 (4–4)	2(1–2)	2(2–3)
PAC SYM score, median (IQR)	1 (0–4)	34 (29–36)^*^	24 (21–30)
GSRS Score, median (IQR)	18.5 (17–24)	61 (59–68)	60 (59–61)
Colonic transit time, median (IQR)	Not performed	4.9 (2.7–5.9) days^*^	1.8 (1.5–2.8) days

* marks difference (*p* < 0.05) between patients with functional constipation and irritable bowel syndrome constipation type. Colonic transit was assessed in 11 patients with FC and 8 patients with IBS‐C

### 3.2 Questionnaires

Scores from the questionnaires are given in Table 1 and showed no difference in the GSRS between the two patients groups but a significant difference in the PAC SYM score. By definition, HC had a low score in both the GSRS and PAC SYM questionnaires. No correlation was found between colonic transit time and the results from the GSRS (Spearman's rho 0.44 and *p* = 0.323) or PAC SYM (Spearman's rho 0.30, *p* = 0.225).

### 3.3 Colonic volumes

The 42 MRI scans were of good quality, allowing estimation of all colonic volumes. However, 17 scans did not include the most distal 2–3 centimeters of the rectum, wherefore both total colonic (colorectal) volume and colonic volume oral to the sigmoid colon were assessed.

**FIGURE 1:**
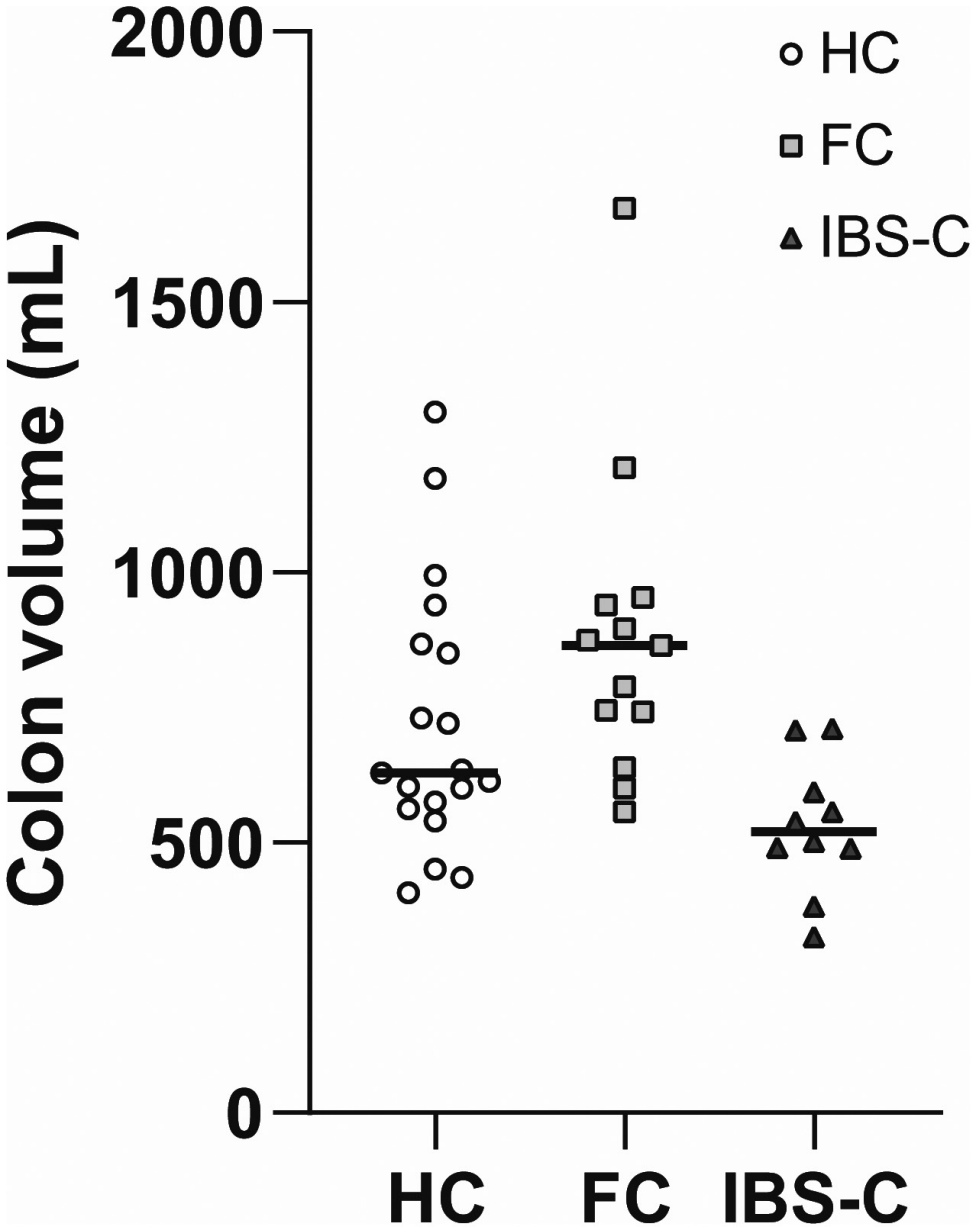
Total colonic volume assessed by Magnetic Resonance Imaging. The medians are given in each group. Colonic volumes of patients with IBS‐C were lower than those of HC and patients with FC. Abbreviations: HC, Healthy controls, FC, Functional constipation, IBS, Irritable bowel syndrome constipation type

**FIGURE 2:**
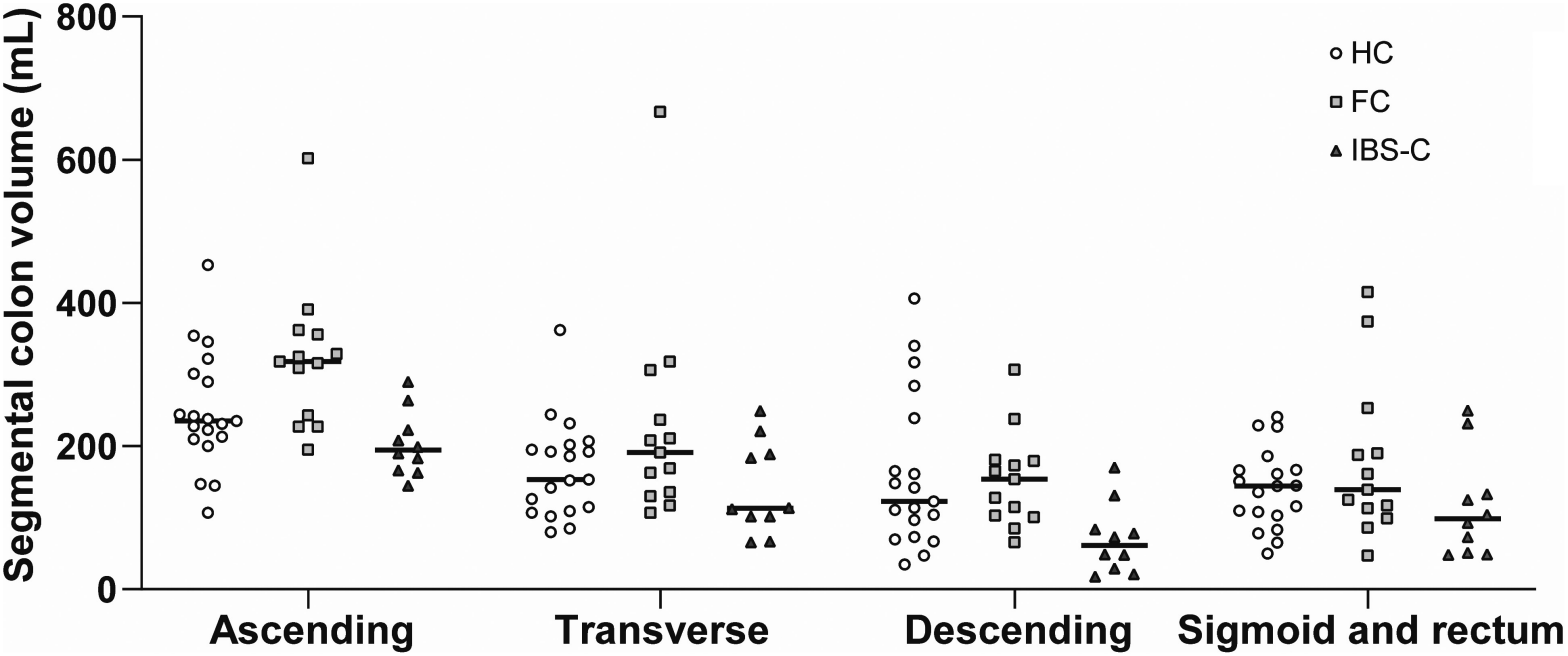
Segmental colonic volume measured by Magnetic Resonance Imaging. The medians are shown in each group. Ascending colonic volume was higher in patients with FC (*p* < 0.01), while patients IBS‐C had a small volume of the descending colon (*p* < 0.02). Comparing the two groups of patients, those with IBS‐C had lower volumes of the ascending colon (*p* < 0.01), the transverse colon (*p* < 0.05) and the descending colon (*p* < 0.01). Abbreviations: FC, Functional constipation. IBS‐C, Irritable bowel syndrome constipation type

Total and segmental colonic volumes are shown in Table 2, Figure 1, and Figure 2. Overall, total colonic volume differed between the three groups (*p* = 0.001) (Figure 1). Patients with FC had a greater colonic volume than patients with IBS‐C and HC. The overall difference between groups remained even if the rectosigmoid colon was excluded from the analysis.

**TABLE 2:** Segmental colonic volumes assessed with magnetic resonance imaging. All data are given in medians. Brackets show interquartile range.

Segmental colonic volumes	Healthy controls (HC)	Functional constipation (FC)	Irritable bowel syndrome constipation type (IBS‐C)
Ascending colon (ml )^†,*^	235 (210–301)	318 (243–356)	195 (166–223)
Transverse colon (ml )^*^	153 (109–202)	191 (136–237)	113 (102–189)
Descending colon (ml )^°,*^	123 (73–239)	154 (103–179)	61 (29–84)
Rectosigmoid colon(ml )	144 (103–167)	139 (113–190)	99 (51–133)
Colon total (ml ) ° *	629 (532–868)	864 (742–940)	520 (489–593)

Note

Abbreviations: Asc: Ascending colon, Tra: Transverse colon, Dsc: Descending colon, Sig: Rectosigmoid colon, HC: Healthy controls, FC: FC. IBS‐C: Irritable bowel syndrome, constipation type.

* mark significant difference (*p* < 0.05) between patients with FC and IBS‐C, ° mark difference (*p* < 0.05) between patients with IBS‐C and HC and † mark (*p* < 0.05) difference between patients with FC and HC

**FIGURE 3:**
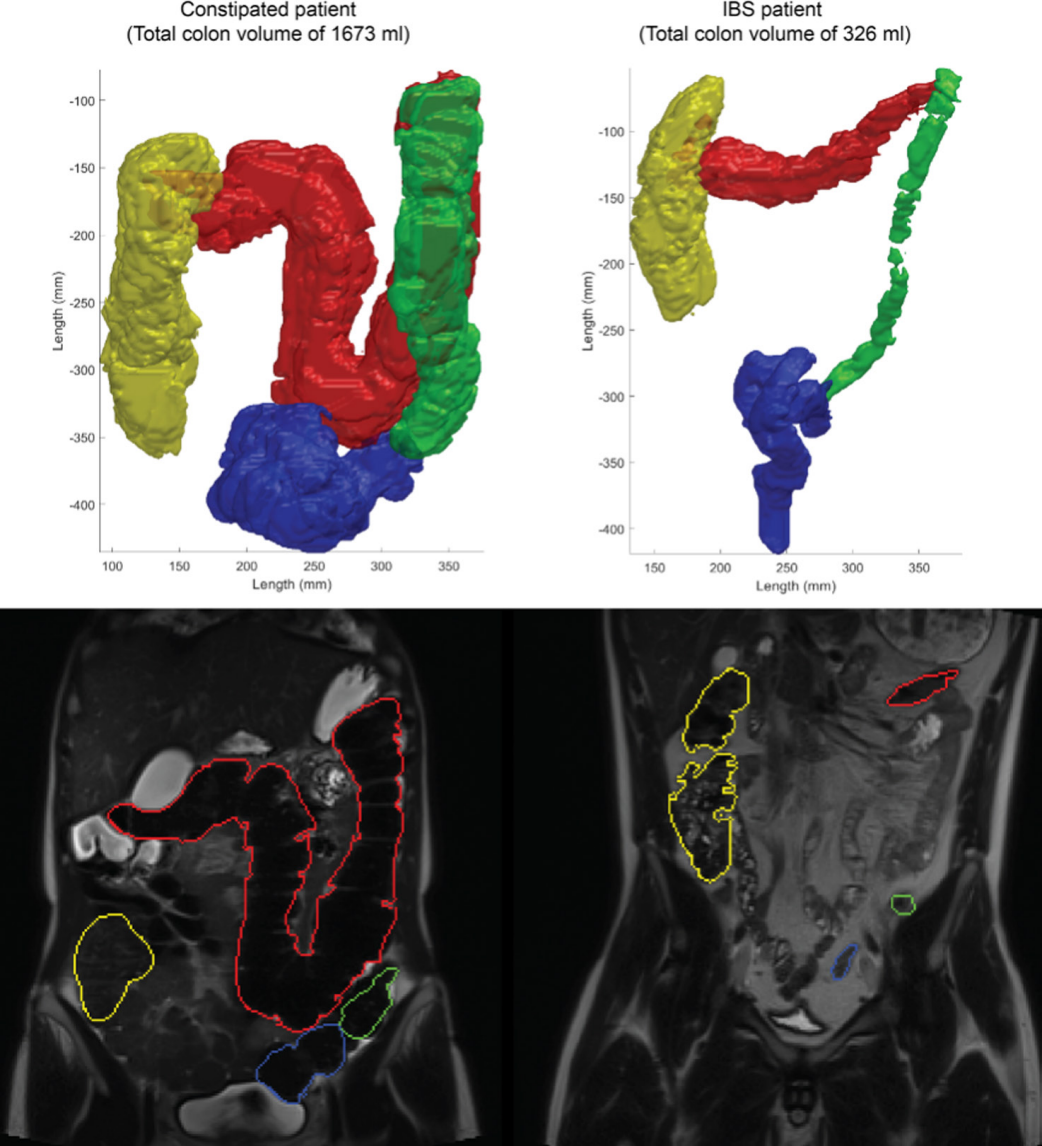
Representative MRI scans (bottom) and volume reconstructions (top) demonstrating the segmental volumes from a patient with functional constipation (to the left) and a patient with irritable bowel syndrome, constipation type (to the right)

Segmental colonic volumes differed between HC and patients (Figures 2 and 3). Thus, ascending colonic volume was higher in patients with FC than in HC (*p* < 0.01), while IBS‐C patients had lower volume than HC in the descending colon (*p* < 0.02). Comparing the two groups of patients, IBS‐C had lower volumes of the ascending colon (*p* < 0.01), the transverse colon (*p* < 0.05) and the descending colon (*p* < 0.01).

We found correlations between colonic transit time and volumes of the ascending colon (Spearman's rho 0.52, *p* = 0.022), the transverse colon (Spearman's rho 0.56, *p* = 0.012), and descending colon (Spearman's rho 0.54, *p* = 0.017). The association with colonic volume of the rectosigmoid was of borderline significance (Spearman's rho 0.39, *p* = 0.097).

### 3.4 Colonic transit times

**FIGURE 4:**
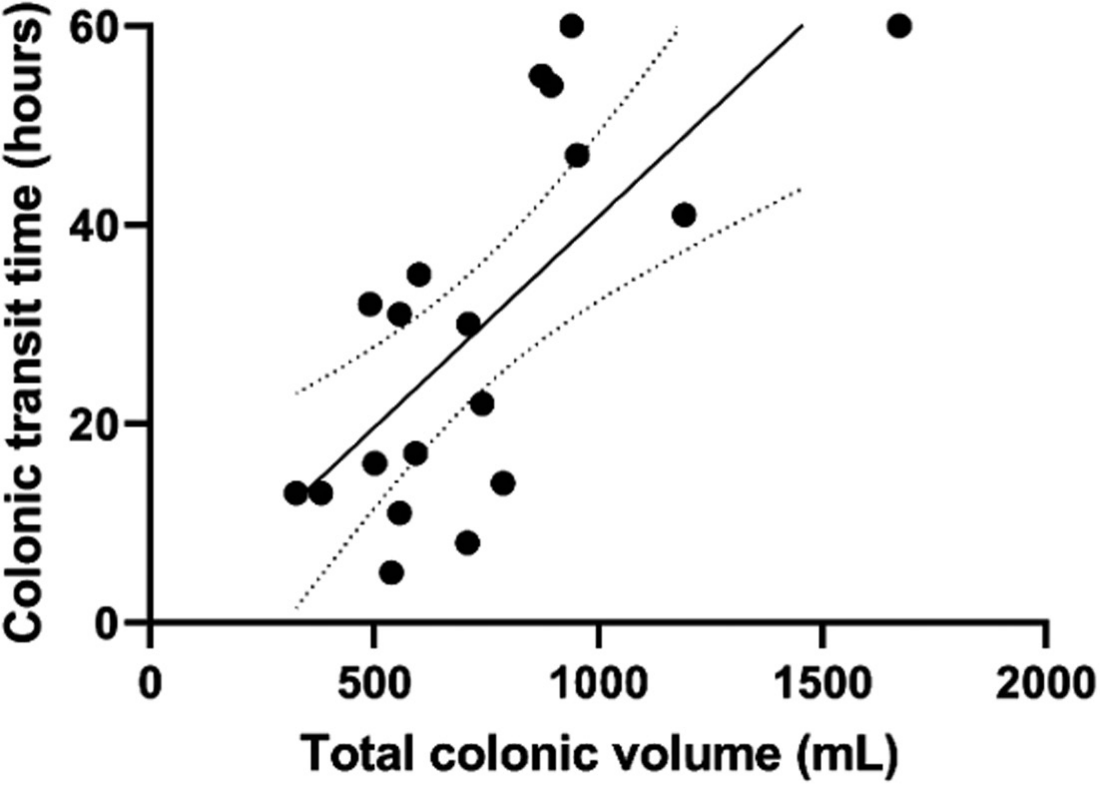
Association between total colonic volume determined with Magnetic Resonance Imaging and colonic transit time assessed with radiopaque makers (Spearman´s rho 0.708, *p* < 0.001). Dotted lines show the 95% confidence interval to the trend line. The radiopaque marker examination was performed in 11 patients with functional constipation and 9 patients with irritable bowel syndrome, constipation type

In patients with FC, the colonic transit time was 4.9 days, 63% longer than in patients with IBS‐C (1.8 days), Table 1. We found a positive correlation between total colonic volume and colonic transit time. (Spearman's rho 0.708, *p* < 0.001), Figure 4.

### 3.5 Stool consistency

Median stool consistency categorized by the BSFS HCs had a higher BSFS score than patients with FC (Table 1). The difference between patients with FC and IBS‐C was of borderline significance (Table 1). Colonic transit times were only available from the two patient groups, we found no association between the total colonic volume and stool consistency (Spearman's rho −0.164, *p* = 0.307) or between stool consistency and colonic transit times (Spearmen's rho −0.39 *p* = 0.097).

## 4 DISCUSSION

Contrary to our hypothesis, the total colonic volumes of patients with FC or IBS‐C were not larger than those of HC, although patients with FC had a higher volume of the cecum and ascending colon. Surprisingly, the total colonic volume of patients with IBS‐C was smaller than in the other groups. Accordingly, patients with IBS‐C had faster colonic transit time compared to patients with FC.

A previous MRI study investigated changes in intestinal volume, motility index, small bowel water content, and gut transit times in response to laxatives in patients with FC or IBS‐C.^17^ Patients with FC had a more voluminously ascending colon than patients with IBS.^17^ Stool consistency did not differ between the two groups.^17^ The present study confirmed those findings and also included HCs. Hence, measurement of the ascending colonic volume, perhaps in combination with the descending colonic volume, may prove a future method to differentiate between patients with FC and IBS‐C.

### 4.1 Objective assessment of constipation

In another MRI study on 25 patients with IBS diarrhea type, the segmental colonic volumes reported were similar to those of the present study.^24^ This indicates that the cause of gastrointestinal symptoms in either type of IBS is not abnormally large distension of the colon. A more likely explanation would be that IBS patients of either type are hypersensitive to colonic distension.^24^, ^25^ This theory was supported by a study showing that 20% of patients with IBS had increased rectal sensation, while another paper concluded that diarrhea‐predominant IBS was associated with rectal hypersensation.^26^, ^27^ Likewise, IBS‐C is associated with rectal hyposensitivity and reduced call for stools.^27^

### 4.2 Transit times

In agreement with a previous studies, we found that patients with FC had significantly longer colonic transit times (median 4.9 days) than those with IBS‐C (median 1.8 days).^17^, ^28^ A recent study in pediatric constipated patients found a positive correlation between whole gut transit times and colonic volume.^15^ Colonic transit times have significant interindividual and day‐to‐day variation. All studies mentioned, including our own, included relatively few patients. It remains unknown whether the same is true for colonic volumes, but studies with robust numbers of patients and controls should be performed before making any firm conclusions.^29^, ^30^, ^31^

### 4.3 Implications for diagnosis and treatment

Patients with symptoms of FC are usually classified into those with prolonged or normal transit constipation. Symptoms can also be caused by evacuation disorders and hyposensitivity of the rectum. Rectal balloon distension or the barostat are useful for evaluating rectal sensory and motor function, while anorectal manometry and defecography help identify evacuation disorders.^27^ The present study was not performed to assess volumetric colon MRI as a diagnostic test of neither FC nor IBS‐C. However, it was striking that patients with IBS‐C had lower colonic volumes than patients with FC and HC. Future studies are needed to determine whether patients with large colonic volumes respond better to laxatives or prokinetics than those with normal or small volumes. If so, assessment of colonic volumes may allow clinicians to stratify patients and thereby guide treatment. In addition, future developments with postprocessing of MRI data will allow separation of colonic content into solid, fluid, and gas.^32^ Such information may be important for evaluating individual patients and the effects of treatment or diet.

### 4.4 Limitations

There are several limitations to our study. Our primary endpoint came out negative, and conclusions based on secondary endpoints should be taken with caution. Patients and HC were allowed to follow their normal daily routines, except for taking laxatives, and we have no information on diet, intake of fluid or physical activity. A more standardized protocol might have given other results, but we chose to study patients under circumstances close to their daily routines.

The relatively low number of subjects studied does not allow firm conclusions to be drawn and results should be considered explorative. Thus, results should be interpreted with caution, and larger studies are needed before clinical use of colonic volumes in clinical practice.

We assessed colonic transit times with radiopaque markers as this method is simple and safe and does not interfere with the MRI. Strictly speaking, the method determines total gastrointestinal (oro‐anal) transit time and not only the colonic transit time. Since most subjects have much longer transit time through the colon than through the stomach and small intestine, total gastrointestinal transit time is often used as a proxy for colonic transit time.

We assessed bowel symptoms by means of the PAC SYM score and found that patients with FC had the most severe symptoms, followed by patients with IBS‐C, while HC were (by nature) symptom‐free. This finding is in contrast to a very large study in which patients with IBS‐C had a higher burden of gastrointestinal symptoms than patients with idiopathic constipation.^3^ Hence, our patients may not represent the wider population of patients with FC or IBS‐C. In Denmark, patients with FC or IBS are mainly treated in primary care, while few are seen by gastroenterologists. We included patients from outpatient clinics at a regional and a university hospital. All were classified according to the ROME IV criteria. Still our cohort represents a selected group of patients, most likely those with more severe or persisting symptoms. As expected, the group of patients with FC were older than those with IBS‐C. It is debatable whether age and colonic transit time are associated, and it remains unknown whether age and colonic volume are so.^8^, ^33^

## 5 CONCLUSION

In conclusion, we found an overall difference between total colonic volumes in patients with FC, IBS‐C, and HC. Compared with HC, patients with FC had larger volumes of the right colon and patients with IBS‐C had smaller colonic volumes than either HC or patients with FC. MRI holds promise as a research tool for evaluating colonic disorders, and this quantitative imaging method may support future development and evaluation of direct mechanisms‐based treatments. However, the number of subjects included in the study was relatively low and results should be considered explorative. Thus, the clinical utility of volume assessment of colonic volumes in patients with FC or IBS‐C remains to be established.

## DISCLOSURES

No competing interests declared.

## AUTHOR CONTRIBUTIONS

Mette W Klinge; Project: (organization, data processing). Statistical analysis (design, data analysis, data processing, interpretation of data, design of figures). Manuscript (writing first draft). Klaus Krogh; Project (conception, organization, recruiting patients, study supervision). Statistical analysis (design, interpretation of data, critical review). Manuscript (writing first draft of manuscript, critical review and revision). Esben Bolvig Mark; Statistical analysis (design, critical review, interpretation of data, processing MRI data, design of figure/imaging). Manuscript (drafting manuscript, review, and critical revision). Asbjørn Mohr Drews, Project (organization, conception). Statistical analysis (critical review, interpretation of data). Manuscript (drafting manuscript, critical review). Lau Brix Project (execution of MRI scans, supervision of data processing). Statistical analysis (critical review, interpretation of data). Manuscript (drafting of manuscript, critical review). Christin Isaksen, Project (execution of MRI scans, supervision of data processing). Statistical analysis (critical review, interpretation of data). Manuscript (drafting of manuscript, critical review). Milda Dedelaité, Project (supervision of data processing, manually control of MRI data). Statistical analysis (critical review). Manuscript (drafting of manuscript, critical review). Jens Brøndum Frøkjær. Project (conception, organization, study design, supervision of data processing). Statistical analysis (design, data interpretation, critical review). Manuscript (drafting of manuscript, critical review). Lotte Vinskov Fynne; Project (conception, organization, execution, recruiting participants, applying for economic foundation). Statistical analysis (design, critical review, interpretation of data). Manuscript (Writing first draft, review, and critical revision).

## References

[1] Belsey J , Greenfield S , Candy D , Geraint M . Systematic review: Impact of constipation on quality of life in adults and children. Aliment Pharmacol Ther. 2010;31:938‐949. 20180788 10.1111/j.1365-2036.2010.04273.x

[2] Rao SSC , Tuteja AK , Vellema T , Kempf J , Stessman M . Dyssynergic defecation: Demographics, symptoms, stool patterns, and quality of life. J Clin Gastroenterol. 2004;38:680‐685. 15319652 10.1097/01.mcg.0000135929.78074.8c

[3] Heidelbaugh JJ , Stelwagon M , Miller SA , Shea EP , Chey WD . The spectrum of constipation‐predominant irritable bowel syndrome and chronic idiopathic constipation : US Survey assessing symptoms, care seeking, and disease burden. Am J Gastroenterol. 2015;110:580‐587. 25781368 10.1038/ajg.2015.67 PMC4424385

[4] Chey WD , Kurlander J , Eswaran S . Irritable bowel syndrome: A clinical review. JAMA. 2015;313:949‐958. 25734736 10.1001/jama.2015.0954

[5] Foundation R . Rome IV questionaire, https://theromefoundation.org/rome‐iv/rome‐iv‐questionnaire/

[6] Stewart WF , Liberman JN , Sandler RS , et al. Epidemiology of constipation (EPOC) study in the United States: Relation of clinical subtypes to sociodemographic features. Am J Gastroenterol. 1999;94:3530‐3540. 10606315 10.1111/j.1572-0241.1999.01642.x

[7] Pare P , Ferrazzi S , Thompson WG , Irvine EJ , Rance L . An epidemiological survey of constipation in Canada: Definitions, rates, demographics, and predictors of health care seeking. Am J Gastroenterol. 2001;96:3130‐3137. 11721760 10.1111/j.1572-0241.2001.05259.x

[8] Mugie SM , Benninga MA , Di Lorenzo C . Epidemiology of constipation in children and adults: A systematic review. Best Pract Res Clin Gastroenterol. 2011;25:3‐18. 21382575 10.1016/j.bpg.2010.12.010

[9] Foundation R . Guidelines‐Rome III diagnostic criteria for functional gastrointestinal disorders. J Gastrointest Liver Dis. 2006;2006(15):307‐312. 17203570

[10] Kim ER , Rhee P‐L . How to interpret a functional or motility test ‐ Colon transit. J Neurogastroenterol Motility. 2012;18:211‐217. 10.5056/jnm.2012.18.1.94 PMC3271260 22323993

[11] Pedersen CE , Møller IJ , Siggaard C , Krogh K . Behandling af kronisk obstipation hos børn – en gennemgang af et Cochranereview. Ugeskr. Læger. 2013;175:1855‐1858. 23937872

[12] Klinge MW , Borghammer P , Lund S , et al. Enteric cholinergic neuropathy in patients with diabetes: Non‐invasive assessment with positron emission tomography. Neurogastroenterol Motil. 2020;31:e13731. 10.1111/nmo.13731 31595630

[13] Nilsson M , Sandberg TH , Poulsen JL , et al. Quantification and variability in colonic volume with a novel magnetic resonance imaging method. Neurogastroenterol Motil. 2015;27:1755‐1763. 26598050 10.1111/nmo.12673

[14] Lam C , Chaddock G , Laurea LM , et al. Distinct abnormalities of small bowel and regional colonic volumes in subtypes of irritable bowel syndrome revealed by MRI. Am J Gastroenterol. 2017;112:346‐355. 27958282 10.1038/ajg.2016.538 PMC5318666

[15] Sharif H , Hoad CL , Abrehart N , et al. Colonic volume changes in paediatric constipation compared to normal values measured using MRI. Diagnostics. 2021;11:1‐9. 10.3390/diagnostics11060974 PMC8226615 34071217

[16] Major G , Murray K , Singh G , et al. Demonstration of differences in colonic volumes, transit, chyme consistency, and response to psyllium between healthy and constipated subjects using magnetic resonance imaging. Neurogastroenterol Motil. 2018;30:1‐11. 10.1111/nmo.13400 30062794

[17] Lam C , Chaddock G , Marciani L , et al. Colonic response to laxative ingestion as assessed by MRI differs in constipated irritable bowel syndrome compared to functional constipation. Neurogastroenterol Motil. 2016;28:861‐870. 26871949 10.1111/nmo.12784 PMC4949702

[18] Sandberg TH , Nilsson M , Poulsen JL , et al. A novel semi‐automatic segmentation method for volumetric assessment of the colon based on magnetic resonance imaging. Abdom Imaging. 2015;40:2232‐2241. 26054979 10.1007/s00261-015-0475-z

[19] Lewis SJ , Heaton KW . Stool form scale as a useful guide to intestinal transit time. Scand J Gastroenterol. 1997;32:920‐924. 9299672 10.3109/00365529709011203

[20] Heaton KW , Radvan J , Cripps H , Mountford RA , Braddon FEM , Hughes AO . Defecation frequency and timing, and stool form in the general population: A prospective study. Gut. 1992;33:818‐824. 1624166 10.1136/gut.33.6.818 PMC1379343

[21] Frank L , Kleinman L , Farup C , Taylor L , Miner P . Psychometric validation of a constipation symptom assessment questionnaire. Scand J Gastroenterol. 1999;34:870‐877. 10522604 10.1080/003655299750025327

[22] Svedlund J , Sjödin I , Dotevall G . GSRS‐A clinical rating scale for gastrointestinal symptoms in patients with irritable bowel syndrome and peptic ulcer disease. Dig Dis Sci. 1988;33:129‐134. 3123181 10.1007/BF01535722

[23] Abrahamsson H , Antov S , Bosaeus I . Gastrointestinal and colonic segmental transit time evaluated by a single abdominal x‐ray in healthy subjects and constipated patients. Scandinavian J Gastroenterol Suppl. 1988;152:72‐80. 10.3109/00365528809095938 3254616

[24] Pritchard SE , Marciani L , Garsed KC , et al. Fasting and postprandial volumes of the undisturbed colon: Normal values and changes in diarrhea‐predominant irritable bowel syndrome measured using serial MRI. Neurogastroenterol Motil. 2014;26:124‐130. 24131490 10.1111/nmo.12243 PMC3995006

[25] Major G , Pritchard S , Murray K , et al. Colon hypersensitivity to distension, rather than excessive gas production, produces carbohydrate‐related symptoms in individuals with irritable bowel syndrome. Gastroenterology. 2017;152:124‐133.e2. 27746233 10.1053/j.gastro.2016.09.062

[26] Camilleri M , McKinzie S , Busciglio I , et al. Prospective study of motor, sensory, psychologic, and autonomic functions in patients with irritable bowel syndrome. Clin Gastroeterol Hepatol. 2008;6:772‐781. 10.1016/j.cgh.2008.02.060 PMC2495078 18456567

[27] Carrington EV , Scott SM , Bharucha A , et al. Expert consensus document: Advances in the evaluation of anorectal function. Nat Rev Gastroenterol Hepatol. 2018;15:309‐325. 29636555 10.1038/nrgastro.2018.27 PMC6028941

[28] Horikawa Y , Mieno H , Inoue M , Kajiyama G . Gastrointestinal motility in patients with irritable bowel syndrome studied by using radiopaque markers. Scand J Gastroenterol. 1999;34:1190‐1195. 10636065 10.1080/003655299750024698

[29] Degen LP , Philips SF . Variability of gastrointestinal transit in healthy women and men. Henry James in Context. 1996;39:299‐305. 10.1136/gut.39.2.299 PMC1383315 8977347

[30] Birkebaek NH , Memmert K , Mortensen J , Dirksen H , Christensen MF . Fractional gastrointestinal transit time: intra‐ and interindividual variation. Nucl Med Commun. 1990;11:247‐252. 2342723 10.1097/00006231-199003000-00003

[31] Haase AM , Gregersen T , Schlageter V , et al. Pilot study trialling a new ambulatory method for the clinical assessment of regional gastrointestinal transit using multiple electromagnetic capsules. Neurogastroenterol Motil. 2014;26:1783‐1791. 25348504 10.1111/nmo.12461

[32] Mark EB , Bødker MB , Grønlund D , Østergaard LR , Frøkjær JB , Drewes AM . MRI analysis of fecal volume and dryness: Validation study using an experimental oxycodone‐induced constipation model. J Magn Reson Imaging. 2019;50:733‐745. 30609164 10.1002/jmri.26628

[33] Metcalf AM , Phillips SF , Zinsmeister AR , MacCarty RL , Beart RW , Wolff BG . Simplified assessment of segmental colonic transit. Gastroenterology. 1987;92:40‐47. 3023168 10.1016/0016-5085(87)90837-7

